# Reasons for discontinuing insulin and factors associated with insulin discontinuation in patients with type 2 diabetes mellitus: a real-world evidence study

**DOI:** 10.1186/s40842-020-00115-2

**Published:** 2021-01-05

**Authors:** Jianmin Wu, Fritha Morrison, Zhenxiang Zhao, Ginger Haynes, Xuanyao He, Ayad K. Ali, Maria Shubina, Shervin Malmasi, Wendong Ge, Xiaomei Peng, Alexander Turchin

**Affiliations:** 1 Eli Lilly and Company, Indianapolis, IN USA; 2 Division of Endocrinology, Brigham and Women’s Hospital, 221 Longwood Avenue, Boston, MA 02115 USA; 3 Harvard Medical School, Boston, MA USA

**Keywords:** Type 2 diabetes, Insulin, Discontinuation, Real-world evidence

## Abstract

**Background:**

Evidence suggests that insulin therapy of patients with type 2 diabetes mellitus (T2DM) is frequently discontinued. However, the reasons for discontinuing insulin and factors associated with insulin discontinuation in this patient population are not well understood.

**Methods:**

We conducted a retrospective cohort study of adults with T2DM prescribed insulin between 2010 and 2017 at Partners HealthCare. Reasons for discontinuing insulin and factors associated with insulin discontinuation were studied using electronic medical records (EMR) data. Natural language processing (NLP) was applied to identify reasons from unstructured clinical notes. Factors associated with insulin discontinuation were extracted from structured EMR data and evaluated using multivariable logistic regression.

**Results:**

Among 7009 study patients, 2957 (42.2%) discontinued insulin within 12 months after study entry. Most patients who discontinued insulin (2121 / 71.7%) had reasons for discontinuation documented. The most common reasons were improving blood glucose control (33.2%), achieved weight loss (18.5%) and initiation of non-insulin diabetes medications (16.7%). In multivariable analysis adjusted for demographics and comorbidities, patients were more likely to discontinue either basal or bolus insulin if they were on a basal-bolus regimen (OR 1.6, 95% CI 1.3 to 1.8; *p* <  0.001) or were being seen by an endocrinologist (OR 2.6; 95% CI 2.2 to 3.0; *p* <  0.001).

**Conclusions:**

In this large real-world evidence study conducted in an area with a high penetration of health insurance, insulin discontinuation countenanced by healthcare providers was common. In most cases it was linked to achievement of glycemic control, achieved weight loss and initiation of other diabetes medications. Factors associated with and stated reasons for insulin discontinuation were different from those previously described for non-adherence to insulin therapy, identifying it as a distinct clinical phenomenon.

**Supplementary Information:**

The online version contains supplementary material available at 10.1186/s40842-020-00115-2.

## Background

Type 2 diabetes mellitus (T2DM) is a highly prevalent chronic disease with serious medical and economic burden [1–3]. The major focus of disease management in patients with T2DM is glycemic control, which might be initially achieved through lifestyle change and treatment with first-line metformin [4]. Due to the progressive nature of this chronic disease, many patients with T2DM eventually will require treatment intensification with other medications, including insulin [5]. Lower incidence of diabetes complications and lower severity of diabetes as well as reduced patient-borne costs have been reported for patients with high adherence to insulin therapy [6, 7]. Despite American Diabetes Association (ADA) guidelines and compelling evidence regarding the benefits of insulin therapy, insulin is underutilized in patients with T2DM. Especially alarming is the number of patients who stop insulin therapy in spite of poorly controlled hyperglycemia [8, 9].

One important aspect of cessation of insulin therapy that has not been well studied is discontinuation of insulin therapy by healthcare providers. While non-adherence to insulin is driven primarily by the patient’s decision not to take the medication, discontinuation of insulin therapy countenanced by the patient’s healthcare provider is a modification of the treatment regimen that may have been prompted by a change in clinical circumstances. However, why insulin therapy is being discontinued is not completely understood. Important aspects of discontinuation of insulin therapy include both the reasons for discontinuation and treatment characteristics that are associated with higher incidence of insulin discontinuation. One reason for this knowledge gap is that most previous analyses of discontinuation of insulin therapy (a.k.a. insulin non-persistence) were restricted to administrative/claims data [10–13]. These data sources can identify patients who stopped taking their medication, but they contain little information about the reasons for discontinuation, and may not be able to differentiate between patient-driven non-persistence and insulin discontinuation recommended by a healthcare provider. Other studies used surveys or interviews to identify the factors and reasons associated with medication non-adherence or discontinuation [14, 15]. However, most of these have focused specifically on insulin non-adherence. Furthermore, the quality and validity of data obtained through these approaches may be affected by selection bias, recall bias and response rates [16, 17]. Finally, the cost, time and other resources involved in interviews may be prohibitive.

Electronic medical records (EMR) present a unique opportunity to evaluate both reasons and factors associated with insulin discontinuation. Many EMR systems allow clinicians to electronically record pertinent clinical information, including reasons for discontinuing a medication. Furthermore, EMR systems also include a variety of data that may influence insulin discontinuation decisions but is not available in claims data, such as vital signs / body mass and laboratory test measurements. EMR systems capture patient information using both structured data (based on controlled vocabularies) and unstructured narrative text. We therefore leveraged analysis of structured and unstructured EMR data to conduct this study to identify reasons for discontinuation of insulin and determine factors associated with insulin discontinuation in patients with T2DM.

## Methods

### Study design

We conducted a retrospective cohort study where we used structured EMR data to study prevalence and factors associated with insulin discontinuation, and a combination of structured EMR data and natural language processing (NLP) analysis of unstructured EMR data to identify reasons for discontinuation of insulin therapy among patients with T2DM. An NLP tool was developed and validated to identify documentation of insulin discontinuation reasons in narrative EMR provider notes.

### Study cohort

Adult patients with T2DM, treated at outpatient primary care or endocrinology practices affiliated with Partners HealthCare between January 1, 2010 and September 30, 2017, were studied. Partners HealthCare is an integrated health care delivery network in eastern Massachusetts that includes Brigham and Women’s Hospital, Massachusetts General Hospital, several community hospitals and multiple affiliated outpatient practices. Patients were included in the analysis if they were at least 18 years old, had diabetes mellitus and were treated with insulin. Patients with T2DM were identified based on any one of the following: a) a T2DM entry on the EMR Problem List; b) at least two ICD9 or ICD10 diagnosis codes for T2DM or c) HbA1c ≥ 6.5% for at least 9 months (to exclude gestational diabetes mellitus (GDM)) with no intervening measurements < 6.5%. The date of the first record of insulin treatment during the study period served as the patient’s index / study entry date. Patients were excluded if they had a) diagnosis of type 1 diabetes; b) diagnosis of GDM and no diagnosis of T2DM; c) diagnosis of secondary diabetes; d) history of chronic pancreatitis or pancreatectomy; e) diagnosis of neonatal diabetes; f) prescriptions for urine ketone strips (as evidence for type 1 diabetes mellitus); g) C-peptide < 0.2 pmol / L; or h) anti-glutamic acid decarboxylase (GAD) or anti-islet antibodies.

### Natural language processing tools development and validation

NLP tool for identification of documentation of insulin discontinuation reasons from narrative EMR documents was developed using Canary (http://canary.bwh.harvard.edu) [18], an open source platform that allows users to design and execute rule-based NLP tools that include the following components: a) tool-specific lexicon (*word classes*, representing semantic groupings); b) a set of grammar rules (*phrase structures*) that describe how a particular concept may be represented in written text as well as related concepts (to exclude false positive matches); and c) output triggers, that define when output is generated. The tools were developed based on a training dataset comprised of 18,900 randomly selected electronic provider notes for patients with T2DM manually annotated by trained clinicians. The tools were then validated against a non-overlapping test set of 2000 manually annotated electronic provider notes. The NLP tool was evaluated at the level of pre-specified discontinuation reason categories (see Table 4 for the list of reason categories). Validation metrics included sensitivity, specificity, positive predictive value (PPV) and F_1_ score (a harmonic mean of sensitivity and PPV). Validation metrics for insulin discontinuation reason categories were calculated for all categories together, as many of the individual categories were not expected to be prevalent enough to allow sufficiently narrow confidence intervals.

### Study measurements

Demographic information, medication information, and laboratory data were obtained from the EMR and internal claims (submitted by the study institutions to the patient’s insurance) data at Partners HealthCare. Patients were followed for 12 months after the index date (follow-up period). Patients who discontinued insulin were identified using structured EMR data as one of the following: a) explicit discontinuation of an insulin record in the EMR without initiation of another insulin medication of the same category (basal or prandial) within 90 days; or b) no prescriptions for insulin in a given category (basal or prandial) for 12 months (in absence of an explicit documentation of insulin discontinuation). This definition was based on the typical prescribing workflow in the U.S. that allows a single prescription to last the patient for up to 12 months (including refills). Therefore, either an explicit deactivation of the insulin record or lack of prescriptions for over the maximum period of time that a single prescription could last would indicate that insulin therapy has been discontinued. If a patient was taking more than one insulin category (e.g. both long- and rapid-acting insulin), discontinuation of either insulin category (e.g. only rapid-acting insulin) without a new prescription in the same category within 90 days was considered to be an insulin discontinuation event. Therefore some insulin discontinuations (e.g. discontinuation of prandial but not basal insulin) represented simplification of the regimen rather than a complete cessation of all insulin therapy. Reasons for insulin discontinuation were identified over the follow-up period based on the combination of EMR medication data and NLP analysis of EMR provider notes. Charlson Comorbidity Index (CCI) was calculated from ICD-9 and ICD-10 diagnosis codes from internal claims data as previously described [16, 17]. Body mass index (BMI) increase was calculated as the difference between the BMI closest to the index date (study entry) and the BMI closest to 12 months after the index date. Patient’s weight change was calculated as % increase in weight from the measurement closest to the index date to the measurement closest to 12 months after the index date. Both were positive if the patient’s body mass increased over the study period. HbA1c decrease was similarly calculated as the difference between the HbA1c measurement closest to the index date and the HbA1c measurement closest to 12 months after the index date. Patient’s baseline encounter frequency was calculated as the number of encounters (represented as EMR notes) at the practice where insulin was prescribed over the 12 months preceding the index date (baseline period). Provider characteristics (gender, specialty and diabetes treatment experience) were identified for the provider who entered the patient’s first insulin record in the EMR as this person was most likely to be the clinician treating the patient’s diabetes. Provider diabetes management experience was calculated as the number of unique patients with diabetes seen by the provider over the baseline period.

### Statistical analysis

Summary statistics were conducted by using frequencies and proportions for categorical variables and using means, standard deviations, and medians for continuous variables. Univariate comparisons were conducted using the t test for continuous variables and chi-square test for categorical variables. A multivariable logistic regression model was used to identify factors associated with insulin discontinuation. The model included patient demographics (age, gender, race, marital status and median household income by zip code); blood pressure, BMI increase, HbA1c, low density lipoprotein cholesterol (LDL), estimated glomerular filtration rate (eGFR); smoking history; history of coronary artery disease (CAD), stroke, peripheral vascular disease (PVD), hypertension (HTN), depression, bipolar disorder or schizophrenia; CCI; history of hypoglycemia; number of diabetes and non-diabetes medications at study entry; number of adverse reactions to non-insulin medications, patient’s encounter frequency in the practice where insulin was prescribed; gender, specialty and diabetes experience of the provider who prescribed insulin; presence of a nurse practitioner in the practice; insulin regimen (e.g. basal or basal-bolus); and commercial vs. internally developed EMR. Vital signs / laboratory tests were ascertained using the most recent measurement in the EMR within a year prior to the index date. If there were no measurements within the year prior to the index date, a measurement closest to the index date within a month after it was used. If neither was available, the variable was considered missing.

The primary multivariable logistic regression analysis of the factors associated with insulin discontinuation included all study patients. Multiple imputation was used to account for missing data in the primary multivariable analysis. As some of the variables (HbA1c, LDL and eGFR) were missing for a particularly large number of patients, we also conducted two sensitivity analyses: a) a multivariable logistic model that did not include variables with large amount of missing data (HbA1c, LDL and eGFR) and b) a multivariable model that included all variables with missing data but only included patients who did not have any missing data in these variables. All statistical analyses were conducted using SAS 9.4 (Cary, NC).

### NLP tool development and evaluation

The Canary insulin discontinuation reasons NLP tool included 220-word classes and 615 phrase structures. Examples of word classes included groups of words representing <*glucose*>, <*low* > and related actions (e.g. <*call*>). These were hierarchically grouped into phrase structures that represented either the concept being sought (e.g. <*glucose* > <*low* > − representing hypoglycemia as a reason for insulin discontinuation) or concepts that were excluded (e.g. <*call > <if* > <*low* > <*glucose*>). Sensitivity of the NLP tool was 76.9% (95% CI ± 6.12%), specificity 99.9% (95% CI ±0.02) and positive predictive value 89.7% (95% CI ± 4.77%); F_1_ score was 0.83.

## Results

### Study population

**Fig. 1:**
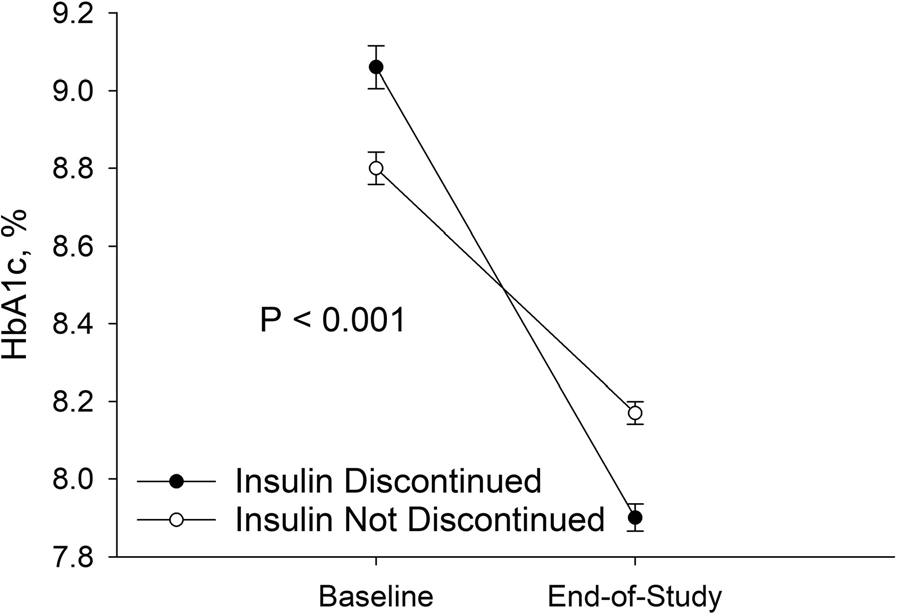
Insulin Discontinuation and Glycemic Control. The figure represents the patients for whom HbA1c measurements were available (1588 out of 2957 patients who discontinued and 2100 out of 4052 patients who did not discontinue insulin therapy at baseline; 2584 out of 2957 patients who discontinued and 3547 out of 4052 patients who did not discontinue insulin therapy at end-of-study). Circles represent mean values. Wisps indicate standard error. Paired t-test was used to determine statistical significance (only for the 3580 patients who had both baseline and end-of-study HbA1c measurements available)

We identified 8744 patients with diabetes mellitus treated with insulin in a primary care or endocrinology clinic affiliated with one of the study institutions. After 1735 patients were removed from the analysis based on the inclusion and exclusion criteria (Table 1), 7009 patients were included in the study. Insulin was discontinued by 2957 (42.2%) study patients. Patients who discontinued insulin lost 0.10% of body weight while patients who did not discontinue insulin gained 0.90% of body weight over the study period (*P* <  0.001). Most (2509 / 84.8%) of insulin discontinuations were identified based on provider orders to stop the medication recorded in the EMR. Patients who had insulin discontinued had a greater decrease in HbA1c over the study period compared to patients who did not (Fig. 1). Majority (71.7%) of patients who discontinued insulin had at least one documented reason for discontinuation; demographic characteristics and comorbidities of patients who had discontinued insulin and those who had reasons for insulin discontinuation documented were similar to the overall study population (Table 2 and Supplemental Table 1).

**Table 1:** Patient flow chart

Criterion	Number of patients in the population after criterion applied
Patients who fulfill inclusion criteria	8744
No evidence of secondary diabetes	7168
No evidence of chronic pancreatitis or pancreatectomy	7072
No evidence of neonatal diabetes	7072
No urine ketone strips prescriptions	7071
No C-peptide < 0.2 pmol / L	7055
No anti-GAD or anti-islet antibodies	7009

**Table 2:**
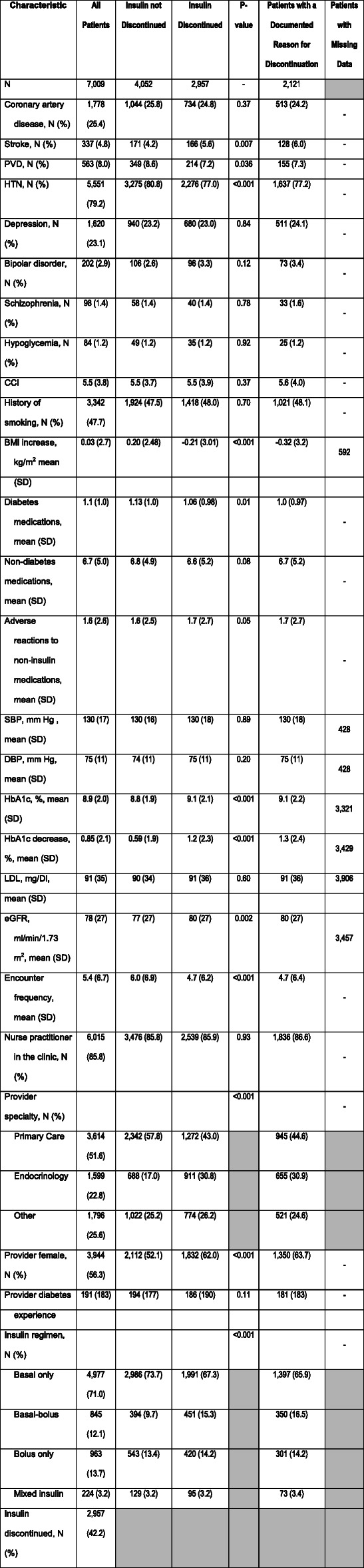
Patient characteristics part I

Cells where the metric is not applicable to the variable are asterisks

-: no missing data for this variable

*BMI* Body mass index, *CCI* Charlson Comorbidity Index, *DBP* Diastolic blood pressure, *eGFR* Estimated glomerular filtration rate, *HbA1C* Hemoglobin A1c, *HTN* Hypertension, *LDL* Low density lipoprotein cholesterol, *PVD* Peripheral vascular disease, *SBP* Systolic blood pressure, *SD* Standard deviation

### Reasons for insulin discontinuation

#### Insulin discontinuation reasons

Reasons for insulin discontinuation were identified in 2121 (71.7%) out of 2957 patients who discontinued insulin. Reasons for insulin discontinuation were recorded in structured EMR data for 950 (44.8%) patients, narrative EMR provider notes for 1754 (82.7%) patients and in both structured data and narrative provider notes for 583 (27.5%) patients. The most common reasons for insulin discontinuation were adequate blood glucose control, achieved weight loss and initiation of non-insulin diabetes medications (Table 3); examples of documentation of the most common insulin discontinuation reason categories are provided in Table 4. Among 2650 patients who discontinued insulin and had BMI data available, patients who did vs. did not have achieved weight loss recorded as one of insulin discontinuation reasons, lost 0.86% vs. gained 0.26% of their body weight over the 12-month study period (*P* = 0.003). The majority (1345 / 63.4%) of patients had multiple reasons for insulin discontinuation recorded. A total of 4861 unique patient-reason combinations were identified in the dataset. The most common combinations of reasons for insulin discontinuation was adequate blood glucose control and achieved weight loss (Supplemental Table 2).

**Table 3:** Distribution of reasons for insulin discontinuation

Reason Category	Number of Events	% Unique Patients	% Unique Reasons
Blood glucose control	1612	76.0	33.2
Weight loss	901	42.5	18.5
Non-insulin diabetes medication started	810	38.2	16.7
Hypoglycemia	585	27.6	12.0
Financial	207	9.8	4.3
Diet	202	9.5	4.2
Decreased food intake	187	8.8	3.9
Patient preference	83	3.9	1.7
Bariatric procedure	60	2.8	1.2
Steroid taper	39	1.8	0.8
Other side effects	17	1.1	0.35
After pregnancy^a^	3	0.19	0.06
Patient cannot manage	2	0.12	0.04
Decreased insulin requirements	1	0.06	0.02
Other	152	9.4	3.1

The denominator for the *% Unique Patients* column is the number of patients (2121) who discontinued insulin with documented reasons. The denominator for the *% Unique Reasons* column is the number of unique reasons (4861) in the analytical dataset. As some patients had more than one reason for insulin discontinuation documented, the number of unique reasons is larger than the number of unique patients. Also as a result of multiple insulin discontinuation reasons being sometimes documented for a single patient, the total in column *% Unique Patients* adds up to more than 100%

^a^Due to postpartum improvement in glycemic control and / or re-initiation of non-insulin diabetes therapy for pre-existing type 2 diabetes

**Table 4:** Examples of documentation of reasons for insulin discontinuation^a^

Reason Category	Example
Blood glucose control	Off her insulin. Her BG is wnl as of late.
	She also stopped taking insulin because her glucose levels have been ranging 90–110.
	BS 80s–130s off insulin.
Weight loss	I discussed that he may not need any insulin at all and this is likely due to his significant weight loss.
	He has continued to work on his diet and keep himself active, has lost 30 pounds since last year, come off of insulin in past couple of weeks per PCP.
	He has been losing weight. He has not been able to eat properly. His blood sugars have dropped and he is having hypoglycemic reactions.
Non-insulin diabetes medication started	Pt. would like to switch to oral meds. Discussed. Dr. will D/C insulin.
	I will replace her mealtime Humalog with Victoza.
	Glucotrol XL 2.5 mg qd was started, replacing insulin injections which he previously used
Hypoglycemia	He has been losing weight. He has not been able to eat properly. His blood sugars have dropped and he is having hypoglycemic reactions.
	Insulin stopped due to hypoglycemia.
Financial	She states she has not been taking any insulin as she could not afford it.
Diet	He has continued to work on his diet and keep himself active, has lost 30 pounds since last year, come off of insulin in past couple of weeks per PCP.
Decreased food intake	He has been losing weight. He has not been able to eat properly. His blood sugars have dropped and he is having hypoglycemic reactions.
Patient preference	Not currently on insulin and she really dislikes giving herself the shots.
Bariatric procedure	F/u s/p gastric bypass surgery. Her Lantus was stopped after the surgery.

^a^Reason categories that constituted > 1% of all insulin discontinuation reasons were included

#### Factors associated with insulin discontinuation

In the multivariable analysis of factors associated with insulin discontinuation (Table 5), there are five factors that were significantly associated with insulin discontinuation across the primary analysis and the sensitivity analyses. Patients were more likely to discontinue insulin if they were being seen by an endocrinologist, had a female provider prescribing insulin, were on a basal-bolus insulin regimen or had a stroke. Patients were less likely to discontinue insulin if their BMI had increased over the 12 months following the initial record of insulin therapy. Sensitivity analysis that did not include laboratory test results (HbA1c, LDL and eGFR) that had a large amount of missing data had results similar to the primary analysis (both analyses included 7009 patients). The results from sensitivity analysis that excluded patients who had any missing data in the laboratory test results (2790 patients were included) showed some variations in several factors, e.g. history of stroke, HbA1c levels and encounter frequency.

**Table 5:** Multivariable analysis of factors associated with insulin discontinuation

Characteristic	Labs Multiply Imputed (7009 patients)		Labs Not Included (7009 patients)		Patients with Missing Labs Not Included (2790 patients)	
	OR (95% CI)	***P***-value	OR (95% CI)	***P***-value	OR (95% CI)	***P***-value
Age	0.99 (0.99–0.99)	0.022	0.99 (0.99–0.99)	0.02	0.99 (0.99–1.01)	0.44
Female	1.1 (0.95–1.2)	0.28	1.1 (0.95–1.2)	0.28	1.02 (0.85–1.2)	0.83
Race						
African-American	0.98 (0.84–1.2)	0.83	0.98 (0.84–1.2)	0.83	0.81 (0.62–1.06)	0.12
Asian	1.1 (0.86–1.5)	0.41	1.1 (0.86–1.5)	0.41	0.98 (0.67–1.4)	0.91
Hispanic	0.95 (0.80–1.1)	0.55	0.95 (0.80–1.1)	0.55	0.93 (0.70–1.2)	0.62
Other	1.1 (0.87–1.3)	0.55	1.1 (0.87–1.3)	0.55	1.1 (0.80–1.4)	0.62
Married	1.01 (0.91–1.1)	0.79	1.01 (0.91–1.1)	0.79	1.02 (0.86–1.2)	0.85
Median household income by zip code	1.00 (1.00–1.00)	0.21	1.00 (1.00–1.00)	0.21	1.02 (1.00–1.1)	0.32
Smoking history	1.01 (0.91–1.1)	0.87	1.01 (0.91–1.1)	0.87	1.1 (0.93–1.3)	0.27
CAD	0.98 (0.86–1.1)	0.78	0.98 (0.86–1.1)	0.78	1.01 (0.82–1.3)	0.92
Stroke	1.3 (1.0–1.7)	0.02	1.3 (1.0–1.7)	0.02	1.7 (1.2–2.5)	0.0017
PVD	0.83 (0.68–1.02)	0.08	0.83 (0.68–1.02)	0.08	0.78 (0.58–1.1)	0.11
HTN	0.96 (0.84–1.1)	0.61	0.96 (0.84–1.1)	0.61	0.84 (0.67–1.1)	0.16
Depression	1.03 (0.90–1.2)	0.70	1.03 (0.90–1.2)	0.70	0.12 (0.97–1.4)	0.10
Bipolar	1.25 (0.91–1.7)	0.17	1.25 (0.91–1.7)	0.17	1.3 (0.83–2.1)	0.24
Schizophrenia	0.84 (0.53–1.3)	0.44	0.84 (0.53–1.3)	0.44	0.75 (0.39–1.5)	0.40
Hypoglycemia	0.99 (0.63–1.6)	0.98	0.99 (0.63–1.6)	0.98	0.66 (0.31–1.4)	0.30
CCI	1.03 (1.01–1.05)	0.003	1.03 (1.01–1.05)	0.003	1.01 (0.98–1.04)	0.62
BMI Increase	0.61 (0.50–0.74)	< 0.001	0.61 (0.50–0.74)	< 0.001	0.57 (0.43–0.77)	0.0003
Number of DM non-insulin meds	0.97 (0.92–1.02)	0.27	0.97 (0.92–1.02)	0.27	0.99 (0.91–1.1)	0.95
Number of non-DM meds	1.01 (1.00–1.03)	0.04	1.01 (1.00–1.03)	0.04	1.01 (0.99–1.03)	0.32
Adverse Reactions to non-insulin meds	1.00 (0.98–1.02)	0.91	1.00 (0.98–1.02)	0.91	0.99 (0.96–1.03)	0.78
SBP	1.00 (0.99–1.00)	0.93	1.00 (0.99–1.00)	0.93	1.00 (0.99–1.01)	0.26
DBP	0.99 (0.99–1.00)	0.99	0.99 (0.99–1.00)	0.99	0.99 (0.99–1.01)	0.90
HbA1c	1.06 (1.02–1.1)	0.005			1.1 (1.05–1.1)	< 0.001
LDL	0.99 (0.99–1.00)	0.71			1.00 (0.99–1.00)	0.98
eGFR	1.00 (0.99–1.00)	0.31			1.00 (0.99–1.01)	0.65
Encounter frequency	0.99 (0.98–0.996)	0.004	0.99 (0.98–0.996)	0.004	0.99 (0.98–1.01)	0.25
Nurse practitioner	0.90 (0.77–1.04)	0.16	0.90 (0.77–1.04)	0.16	0.69 (0.44–1.1)	0.10
Provider specialty						
Endocrinology	2.6 (2.2–3.0)	< 0.001	2.6 (2.2–3.0)	< 0.001	2.0 (1.6–2.6)	< 0.001
Other	1.3 (1.1–1.5)	< 0.001	1.3 (1.1–1.5)	< 0.001	1.1 (0.88–1.4)	0.44
Provider female	1.2 (1.1–1.4)	< 0.001	1.2 (1.1–1.4)	< 0.001	1.2 (1.00–1.4)	0.044
Provider diabetes experience^a^	0.93 (0.90–0.96)	< 0.001	0.93 (0.90–0.96)	< 0.001	0.97 (0.93–1.02)	0.24
Insulin regimen^b^						
Basal-bolus	1.6 (1.3–1.8)	< 0.001	1.6 (1.3–1.8)	< 0.001	1.5 (1.1–2.0)	0.003
Bolus only	1.2 (1.04–1.4)	0.01	1.2 (1.04–1.4)	0.01	1.2 (0.91–1.5)	0.22
Mixed	1.2 (0.92–1.6)	0.16	1.2 (0.92–1.6)	0.16	1.3 (0.88–2.0)	0.18
Commercial EMR	0.53 (0.40–0.71)	< 0.001	0.53 (0.40–0.71)	< 0.001	0.68 (0.45–1.04)	0.08

*Labs Multiply Imputed* represents the model (primary analysis) where all patients were included and missing HbA1c, LDL and eGFR data were accounted for by multiple imputation

*Labs Not Included* represents the model where all patients were included but HbA1c, LDL and eGFR variables were not included in the model

*Patients with Missing Labs Not Included* represents the model where patients who missing either HbA1c, LDL or eGFR data were not included in the model (2790 patients were included)

^a^Per 100 patients seen annually

^b^Compared to basal insulin regimen

## Discussion

This population-based analysis leveraging EMR data of over 7000 patients with T2DM showed that many – over a third – discontinued insulin therapy. Among the patients with documented reasons for discontinuation of insulin, the most common reasons were improving blood glucose control, achieved weight loss and initiation of non-insulin diabetes medications. This is the first study, to our knowledge, that leveraged both structured and unstructured EMR data to understand insulin discontinuation.

The most common reason for insulin discontinuation was good blood glucose control. Second (achieved weight loss) and third (non-insulin diabetes medication started) most common reasons for insulin discontinuation were likely related to blood glucose control as well: achieved weight loss leads to a decrease in insulin resistance and ultimately to lower blood glucose levels and insulin requirements [19], while initiation of additional non-insulin medications may achieve improved blood glucose control on its own, obviating the need for insulin. Consistent with this explanation, these three reasons were commonly reported together. A typical example was a middle-aged patient whose diabetes was diagnosed when he was admitted to the hospital with a severe infection (Fournier gangrene). The patient was initially started on basal-bolus regimen. However, as his infection resolved and he improved his diet and lost weight, his blood glucose levels decreased and he was able to discontinue prandial insulin (continuing on a lower dose of basal insulin). At the population level, the finding of a greater decrease in HbA1c among patients whose insulin was discontinued was also consistent with this explanation. Adverse reactions to insulin, such as hypoglycemia, played a smaller role. In a number of cases hypoglycemia was reported together with good blood glucose control and may have been an unintended consequence of the overall lowering of blood glucose levels that ultimately enabled insulin discontinuation. Financial difficulties were reported as a reason for insulin discontinuation for < 10% of patients. This may reflect the fact that Massachusetts, where the study was conducted, has very high penetration of health insurance and this finding may not generalize to the rest of the U.S.

There were several patient and provider characteristics that were strongly associated with insulin discontinuation. The strongest factor for discontinuation of insulin therapy was treatment by an endocrinologist. This may have been due to more time and resources available to endocrinologists to provide lifestyle counseling and / or to their early adoption (and thus greater propensity to prescribe) novel non-insulin diabetes medications. Either of these approaches could lead to improvement in glycemic control and discontinuation of insulin. Female providers were also more likely to discontinue insulin. This could have been a residual effect of specialty – most endocrinologists at Partners HealthCare are women. Patients were more likely to have insulin discontinued if they were treated with a basal-bolus regimen. This could reflect the fact that more complicated insulin regimens can be more challenging to manage, and that could ultimately prompt insulin discontinuation. Patients whose BMI had risen after study entry were less likely to discontinue insulin. This finding could have been confounded: insulin therapy results in weight gain and therefore weight gain could have reflected the fact that treatment with insulin was continued.

Consistent with the objective of the investigation to analyze a distinct clinical phenomenon of insulin discontinuation, factors associated with and reasons for insulin discontinuation identified in our study differed significantly from those previously identified for insulin non-adherence. For example, while previously published studies reported strong associations of age and gender with non-adherence [20–22], neither was related to insulin discontinuation in our findings. Similarly, practical and logistical barriers commonly cited by patients as the reasons for insulin non-adherence [20, 21, 23] were not identified as the reasons for insulin discontinuation in our study.

This analysis of insulin discontinuation by providers was enabled by EMR data. Both claims and EMR data have their strengths and weaknesses [24]. One important distinction of EMR data that was particularly pertinent to the present study is that it contains information on prescriber orders. Claims data allows analysis of whether the patient is taking the medication or not (based on the assumption that patients are unlikely to purchase medications, often incurring copayments, if they are not taking them). However, it is impossible to determine from the claims data whether cessation of medication consumption by the patient was recommended by their healthcare provider. On the other hand, EMR data includes information on prescriber medication orders to both initiate and to discontinue medications. In particular, in our dataset almost all (nearly 85%) of insulin discontinuations were identified based on EMR provider orders to stop the medication. This allowed the present analysis to focus on a previously unexplored aspect of diabetes therapy – insulin discontinuation by healthcare providers.

Most reasons for insulin discontinuation were recorded in narrative provider notes and required NLP technology for their identification. This finding illustrates the opportunities accorded by the increasing availability of multiple streams of EMR data, including narrative electronic documents, combined with the advancements in artificial intelligence technologies that allow population-scale analyses of these data. In particular, natural language processing has shown increasing promise in allowing quantification of previously poorly understood clinical phenomena and their effects on patient outcomes [25–27]. The present study continues to demonstrate the power of this approach by leveraging natural language processing provide initial data on discontinuation of insulin therapy by healthcare providers.

This analysis had a number of strengths. It included a large sample of patients, the majority of whom were treated in a primary care setting, just as most patients with diabetes in the U.S. are. While relatively few patients had reasons for insulin discontinuation recorded in the traditionally used “structured” EMR data (medication lists, adverse medication reaction lists, etc.), this project drew on a novel information source – narrative electronic provider notes. It leveraged validated natural language processing technology to identify reasons for insulin discontinuation from a much larger number of patients than would have been possible either using structured EMR data alone (due to missing data) or manual record review (due to the time and costs involved in reviewing records of thousands of patients). This project therefore represents an advance in clinical research made possible by modern technology.

The findings of this project have to be interpreted in light of its limitations. The analysis was limited to a single integrated healthcare delivery system in Eastern Massachusetts; therefore, the results may not be generalizable to the rest of the U.S. Patients were only followed for 12 months; hence the findings may not be applicable to insulin discontinuation later in the treatment course. Information on the patients’ duration of diabetes was not available for analysis. Some of the patients who had discontinuation of insulin therapy established based on lack of insulin prescriptions for > 12 months may not have, in fact, discontinued insulin; therefore, the rate of insulin therapy discontinuation could have been overestimated. While the accuracy of the natural language processing tools was high, it was not perfect. Consequently, some insulin discontinuation reasons could have been missed and others misidentified. Furthermore, some insulin discontinuation reasons may not have been documented in the EMR at all. Some of the episodes of insulin discontinuation analyzed in the study, while recorded by healthcare providers, may have reflected patient-driven non-adherence. However, based on our clinical experience, patients who are not adherent to their medication that their provider feels is indicated for them seldom have it discontinued in their EMR record – consistent with the finding that patient preference was not commonly cited as a reason for insulin therapy discontinuation in the study data. There was a large amount of missing laboratory data; however, analyses that did not include variables with large amounts of missing data and analyses that imputed missing data had very similar results. Finally, as an observational study, the analysis of factors associated with insulin discontinuation could not identify causal relationships but only associations.

## Conclusions

In summary, this study has confirmed that discontinuation of insulin therapy countenanced by healthcare providers is a distinct clinical phenomenon whose risk factors and reasons are different from the more widely studied medication non-adherence. While many patients discontinued insulin therapy, in most cases discontinuation appeared appropriate. Frequently it was made possible by patients achieving blood glucose control by alternative means, either lifestyle changes, additional non-insulin diabetes medications, or both. Adverse reactions to insulin (e.g. hypoglycemia or weight gain) and financial difficulties played a smaller role than blood glucose control (in an area with a high penetration of health insurance). Many of the factors associated with insulin discontinuation identified in the study (e.g. treatment by an endocrinologist, who may be more likely to use new non-insulin diabetes medications, or a basal-bolus insulin regimen that can be more challenging to manage) were consistent with the overall findings.

## Supplementary Information

**Additional file 1: Supplemental Table 1.** Patient Characteristics Part II. **Supplemental Table 2.** Most Common Combination of Reasons for Insulin Discontinuation.

## Data Availability

The datasets generated during and/or analyzed during the current study are not publicly available due to the institutional patient privacy policy but are available from the corresponding author upon a reasonable request.

## Abbreviations

ADA: American Diabetes Association
BMI: Body mass index
CAD: Coronary artery disease
CCI: Charlson Comorbidity Index
CI: Confidence interval
DBP: Diastolic blood pressure
eGFR: Estimated glomerular filtration rate
EMR: Electronic medical record
GAD: Glutamic acid decarboxylase
GDM: Gestational diabetes mellitus
ICD: International classification of diseases
HTN: Hypertension
LDL: Low-density lipoprotein
NLP: Natural language processing
PVD: Peripheral vascular disease
SBP: Systolic blood pressure
SD: Standard deviation
T2DM: Type 2 diabetes mellitus
