# Supplementary material for: Reasons for discontinuing insulin and factors associated with insulin discontinuation in patients with type 2 diabetes mellitus: a real-world evidence study
Source: Clin Diabetes Endocrinol. 2021 Jan 5;7:1. doi: 10.1186/s40842-020-00115-2 (PMC7786496; doi:10.1186/s40842-020-00115-2)
Supplement: Supplementary file 1 — Additional file 1: Supplemental Table 1. Patient Characteristics Part II. Supplemental Table 2. Most Common Combination of Reasons for Insulin Discontinuation. [file 40842_2020_115_MOESM1_ESM.docx]

**APPENDIX**

Supplemental Table 1. Patient Characteristics Part II.

| **Characteristic** | **All Patients** | **Insulin not Discontinued** | **Insulin Discontinued** | **P-value** | **Reason for Discontinuation Documented** | **Patients with Missing Data** |
| --- | --- | --- | --- | --- | --- | --- |
| N | 7,009 | 4,052 | 2,957 | - | 2,121 |  |
| Age, mean (SD) | 60.2 (13.4) | 60.7 (12.9) | 59.5 (14.0) | <0.001 | 59.1 (14.2) | - |
| Female, N (%) | 3,314 (47.3) | 1,890 (46.6) | 1,424 (48.2) | 0.21 | 1,054 (49.7) | - |
| Race, N (%) |  |  |  | 0.19 |  | - |
| African-American | 966 (13.8) | 564 (13.9) | 402 (13.6) |  | 309 (14.6) |  |
| Asian | 269 (3.8) | 147 (3.6) | 122 (4.1) |  | 91 (4.3) |  |
| Hispanic | 787 (11.2) | 484 (11.9) | 303 (10.3) |  | 234 (11.0) |  |
| White | 4,426 (63.2) | 2,535 (62.6) | 1,891 (64.0) |  | 1,313 (61.9) |  |
| Other | 561 (8.0) | 239 (8.1) | 239 (8.1) |  | 174 (8.2) |  |
| Married, N(%) | 3,653 (52.1) | 2,087 (51.5) | 1,566 (53.0) | 0.23 | 1,118 (52.7) | - |
| Median income by zip code, $10K, mean (SD) | 69.7 (26.3) | 68.9 (26.0) | 70.8 (26.7) | 0.004 | 70.6 (26.7) | 258 |
| Commercial EMR, N (%) | 881 (12.6) | 429 (10.6) | 452 (15.3) | <0.001 | 396 (18.7) | - |

-: no missing data for this variable

Cells where the metric is not applicable to the variable are grayed out

Supplemental Table 2. Most Common Combination of Reasons for Insulin Discontinuation

| **Reason Combination** | **Patients (%)** |
| --- | --- |
| N | 2,121 |
| Blood glucose control + weight loss | 157 (7.4) |
| Blood glucose control + weight loss + non-insulin diabetes medication | 135 (6.4) |
| Blood glucose control + non-insulin diabetes medication | 121 (5.7) |
| Blood glucose control + weight loss + non-insulin diabetes medication + hypoglycemia | 87 (4.1) |
| Blood glucose control + hypoglycemia | 86 (4.1) |
